# Do the Impacts of Mentally Active and Passive Sedentary Behavior on Dementia Incidence Differ by Physical Activity Level? A 5-year Longitudinal Study

**DOI:** 10.2188/jea.JE20210419

**Published:** 2023-08-05

**Authors:** Yuta Nemoto, Shinichiro Sato, Yoshinori Kitabatake, Noriko Takeda, Kazushi Maruo, Takashi Arao

**Affiliations:** 1 Research Team for Social Participation and Community Health, Tokyo Metropolitan Institute of Gerontology, Tokyo, Japan; 2 Faculty of Health Sciences, University of Human Arts and Sciences, Saitama, Japan; 3 Department of Health Sciences, Saitama Prefectural University, Saitama, Japan; 4 Center for Promotion of Higher Education, Kogakuin University, Tokyo, Japan; 5 Faculty of Medicine, University of Tsukuba, Ibaraki, Japan; 6 Meiji Yasuda Life Foundation of Health and Welfare, Tokyo, Japan

**Keywords:** exercise, intellectual activity, recreational activity, dementia onset, competing risk

## Abstract

**Background:**

It has not been determined whether mentally active sedentary behavior (MASB) and passive sedentary behavior (PSB) differentially affect cognitive function and whether these associations differ according to physical activity (PA) level. We examined the comparative impacts of MASB and PSB on dementia onset and aimed to understand whether the associations differed by PA level.

**Methods:**

We conducted a 5-year longitudinal study involving all community-dwelling older adults in a rural area in Japan (*n* = 5,323). Dementia onset was examined using long-term care insurance data. PA was evaluated using the International Physical Activity Questionnaire and categorized as low (<2.5 metabolic equivalent of task [MET]-h/week), moderate (2.5–16.0 MET-h/week), or high (≥16.0 MET-h/week). We also assessed PSB (TV-watching time; <1 h/day, 1–3 h/day, ≥3 h/day) and MASB (Book-reading time; <10 min/day, 10–30 min/day, ≥30 min/day). To examine the associations of MASB and PSB with dementia onset, we performed the Fine-Gray models accounting for competing risk of death.

**Results:**

During the follow-up period, 606 (11.4%) participants developed dementia. MASB was independently associated with a lower risk of dementia; the magnitude of the impact was significant at higher PA levels. There was no association between PSB and developing dementia across all PA levels. Furthermore, dementia risk for individuals with high PA levels and moderate or high MASB levels was approximately 60% lower than those with low PA levels and low MASB.

**Conclusion:**

Providing interventions to promote MASB, which reduces dementia risk, and PA, which increases MASB’s effect on dementia incidence, can be beneficial in delaying or preventing dementia onset.

## INTRODUCTION

The global number of dementia patients more than doubled from 1990 to 2016.^1^ Growing evidence has suggested that modifying risk factors might prevent or delay up to 40% of dementia incidence.^2^ Many studies have investigated the effects of physical activity (PA) on cognitive function^3^ and explored the potential mechanisms.^4^ PA increases cerebral blood flow, which increases brain-derived neurotrophic factor, enhances synaptic strength,^5^ and increases hippocampal capacity.^6^

Sedentary behavior (SB) refers to any waking activity characterized by an energy expenditure of ≤1.5 metabolic equivalence in a seated or reclining posture and is conceptually different from the lack of PA. It is associated with an increased risk of health-related outcomes, such as obesity, hypertension, and diabetes, independent of PA level.^7^^–^^9^ As these diseases are risk factors for dementia onset, SB can, arguably, increase the risk of dementia incidence.^10^^,^^11^

However, several questions about the association of SB with dementia remain unclear. First, the impact of different SB types, such as mentally active sedentary behavior (MASB), which requires cognitive effort, and passive sedentary behavior (PSB), which primarily involves more-passive mental activity,^12^ on dementia onset remains uncertain. Although several systematic reviews have suggested that SB harms cognitive function,^13^ other studies have suggested that individuals with high SB had higher cognitive function.^14^^,^^15^ Previous studies have also revealed that the effect of SB on depression^12^^,^^16^ or cognitive function^17^^–^^20^ differs by type of SB. Therefore, the impact on dementia onset of MASB and PSB may differ. Second, it is uncertain whether PA modifies the association between SB and dementia. Health behaviors, including PA and SB, are interrelated and thus may synergistically affect dementia outcomes.^21^ PA attenuates the impacts of SB on depression,^22^ all-cause mortality,^23^ and cardiovascular disease mortality.^24^ Similarly, PA may modify the effects of SB on dementia onset. Additionally, because multicomponent interventions are necessary to achieve optimal preventive effects on dementia,^25^ identifying the combined impact of PA and SB on dementia onset is required. Third, previous studies that examined the association of PA or SB with dementia did not consider participants who died without dementia. In studies that enroll older populations, competing risks of death cause an overestimation of the effect of PA or SB on dementia risk.^26^

This study aimed to examine the associations between MASB or PSB and dementia onset, and to determine whether these associations differ by PA level. To further identify the associations, a competing risk model was performed to account for dementia-free death.

## METHODS

### Study design and participants

This longitudinal study was conducted over 5 years among community-dwelling older adults living in a rural region. The study location was Tsuru City, Yamanashi, Japan, which had a population of 31,663 and an aging rate of 25.3% in 2016. The eligible participants were all residents aged ≥65 years at baseline (*n* = 8,011). We excluded any participant who had received long-term care insurance (LTCI) services (ie, required frequent assistance with daily living). In total, 6,677 older adults were included in the baseline survey. A self-reported questionnaire was administered in January 2016. Follow-up data were obtained for up to 5 years for those who completed the survey; that is, until the date of dementia onset, the date of dementia-free death, their relocation out of the study area, or the end date of the study (December 31, 2020). Those who applied for LTCI before the baseline survey were excluded from the analysis.

Informed consent was obtained from every participant, and ethical approval was granted by the ethics committee of Waseda University (Approval number: 2015-218).

### Measurements

#### Dementia onset

The incidence of dementia was assessed using LTCI data.^27^^,^^28^ The two-step evaluation was performed when participants applied for LTCI. First, health professionals conducted an on-site assessment using a standardized format.^28^^,^^29^ The participants were categorized into four care needs levels. Level I means that the individual had dementia symptoms but maintained an independent daily life. Level II indicates that the individual showed some symptoms and behaviors relating to dementia that caused difficulties in daily life and communication. Levels III and IV indicate that the individual had dementia-related symptoms as those in Level II but required more frequent care. Additionally, each participant’s home physician also assessed their care needs level based on their medical conditions and physical and cognitive function.^30^ Second, the Care Needs Certification Board, consisting of doctors, nurses, and caseworkers, made the final decision about participants’ care needs levels based on an on-site assessment and the primary physicians’ reports. Levels II to IV were defined as “developing dementia,” which correlated strongly with Mini-Mental State Exam scores.^31^

The evaluation process took approximately 30 days, but the duration varied from 3 to 106 days; therefore, the date on which a participant applied for LTCI was defined as dementia onset if they were evaluated as developing dementia.

#### Physical activity

PA was evaluated with the International Physical Activity Questionnaire Short Version.^32^ Items solicited information about the frequency and duration of vigorous-intensity PA (VPA), moderate-intensity PA excluding walking (MPA), and brisk walking in an average week. This measure reportedly has acceptable reliability and validity among Japanese older adults.^32^ The weekly metabolic equivalent of task (MET-h/week) was computed by summing of VPA (8.0 METs), MPA (4.0 METs), and brisk walking (3.3 METs).^33^ Because the prevalence of higher PA levels was low compared with those reported in a previous meta-analysis,^23^ we classified PA into three levels, not four. The categories were: “low” or <2.5 MET-h/week, “moderate” or 2.5–16.0 MET-h/week, and “high” or >16.0 MET-h/week.

#### Sedentary behavior

SB was assessed based on measurements used in previous studies.^18^^,^^34^ Participants were asked to report the duration of their TV-viewing, computer use, and book- or newspaper-reading in an average day at baseline. In this study, the rate of missing data for computer use time was high (65.9%), owing to the low prevalence of computer users. Thus, computer use time was excluded from the analysis. We defined TV-viewing as PSB and reading books or newspapers as MASB.^12^

In alignment with the previous study,^23^ the amount of SB time spent watching TV was classified into three groups: <1 h/day (low), 1–3 h/day (moderate), and >3 h/day (high); for reading books and newspapers, the categories were <10 min/day (low), 10–30 min/day (moderate), and >30 min/day (high).

#### Covariates

Baseline demographic variables and health status were evaluated as covariates. The demographic variables were sex, age, years of education (<13 years or ≥13 years), marital status (married or divorced/widowed/single), living status (living alone or living with others), and employment status (employed or unemployed). Health status included self-rated health (good or poor), self-reported medical conditions (stroke, diabetes, or hypertension), and frailty. We employed the Kihon checklist to evaluate frailty.^35^ It covers mobility, nutrition, social activity, cognitive function, depression, and oral function. Its total score ranges from 0 to 25, and those with ≥8 points were classified as frail.^36^

### Sample size estimation

Because eligible participants of this study were all older residents aged ≥65, we did not carry out the sample size estimation beforehand. For reference, we conducted the post-hoc sample size estimation without considering multiplicity of statistical tests. Guided by previous studies,^37^^–^^39^ the estimation was based on dementia incidence rates (10% in 5 years), dementia-free death or dementia onset (15%), the correlation between these events (*r* = 0.5), and the hazard ratios for one parameter of interest (0.70 or 1.43). With statistical significance set at 0.05 and power exceeding 80%, the minimum sample size was 5,000 participants. The estimation was performed using the R package powerSurvEpi.^40^^,^^41^

### Statistical analysis

To examine differences in the characteristics between those who developed dementia and those who did not develop dementia, we conducted the independent *t*-test for numerical variables and the chi-square test for categorical variables.

We computed the incidence rate of dementia for each PA and SB category per 1,000 person-years. The cumulative incidence rate was estimated using the cumulative incidence function (CIF) with dementia-free death as a competing risk. The date of death was obtained from the resident registration system.

To identify the impact of PA, MASB, and PSB on the incidence of dementia in the presence of competing risk, we performed the Fine-Gray model. We estimated subdistribution hazard ratio (sdHR) and 95% confidence interval (CI) using the R package cmprsk.^42^ Previous studies have suggested that the sdHR can describe the direction of the effect of PA and SB on the CIF, and that the sdHR could then be interpreted as odds ratios for the CIF if the probability of dementia onset is <0.2.^43^ The incidence rate of dementia in this study was 11.4%; we interpreted the sdHR as the odds ratio for the CIF.

We conducted three analyses using the Fine-Gray model. For all the analyses, model 1 was adjusted for demographic variables and health status, and model 2 was additionally adjusted for other behavioral factors. First, we performed the analyses that included PA or SB as the independent variable to assess the independent impact on dementia onset. Second, we conducted stratified analyses to examine the association of MASB or PSB with dementia at each PA level. Third, we created the combination of PA and MASB/PSB and conducted the analysis that included the combination as the independent variable to examine the collective impact of PA and SB on dementia onset. As the preliminary analysis, we performed sex-stratified analyses. The results showed that, while there were sex differences in PA and MASB, the associations of PA and MASB with dementia onset were similar for both sexes (eTable 1). Therefore, we highlighted the results of the analysis, including all participants.

Two sensitivity analyses were performed to confirm the robustness of the associations of PA, MASB, and PSB on dementia onset. First, to reduce the possibility of reverse causation, we excluded participants who developed dementia within 1 year from the baseline survey. Second, since the novel coronavirus disease 2019 (COVID-19) outbreak affected participants’ lifestyles and health statuses, we analyzed the data collected from baseline to the date of the first COVID-19 state of emergency declared in Japan (April 16, 2020).

The missing rate in each analysis model ranged from 30.0% to 34.7%. For missing information, we used the R package mice^44^ to perform multiple imputations by chained equations, assuming missing at random. Fifty data sets were created, and the combined results of each data set to obtain the estimates.

All analyses were conducted using R 3.6.3 (R Foundation for Statistical Computing, Vienna, Austria).

## RESULTS

### Baseline characteristics

**Figure 1.**
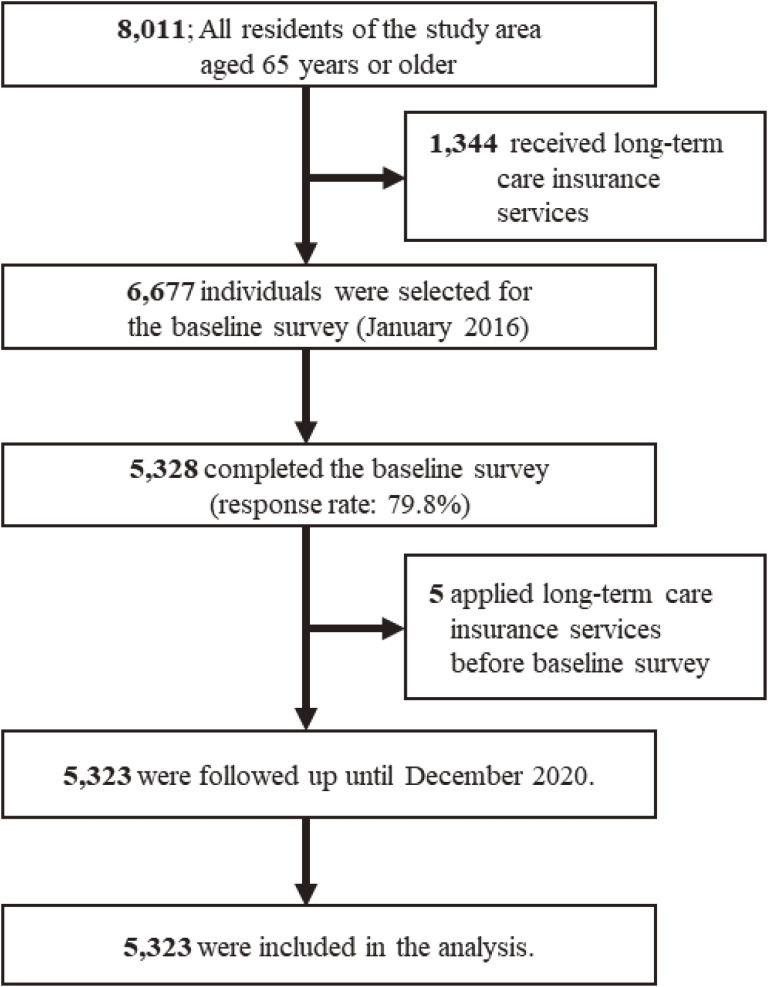
Flow chart of the study

Of the 6,677 eligible participants, 5,328 older adults completed the baseline survey (response rate: 79.8%), and a total of 5,323 individuals were included in the analyses (Figure 1).

The baseline characteristics are shown in Table 1. The mean age for all participants was 74.7 years; 45.5% were males, 38.0% reported low PA, 22.3% reported low MASB, and 28.4% were in the high PSB category. In the 5-year follow-up period, 606 older adults (11.4%) developed dementia. There were significant differences in demographic variables, health status, PA, and MASB at baseline between those who developed dementia and those without dementia (Table 1).

**Table 1.** Baseline characteristics of study participants (*n* = 5,323)

Variables	Total	Developed dementia (11.4%)	Did not develop dementia (88.6%)	Group difference
Age, years, mean (SD)	74.7 (6.9)	81.5 (6.5)	73.8 (6.4)	<0.001
Sex, males, %	45.5	41.7	46.0	0.06
Years of education <13 years, %	85.8	89.0	85.4	0.03
Marital status, married, %	69.9	52.5	72.1	<0.001
Living status, living alone, %	13.3	18.7	12.6	<0.001
Employment status, paid workers, %	27.5	8.8	29.9	<0.001
Self-rated health, good, %	79.8	67.3	81.4	<0.001
BMI, kg/m^2^, mean (SD)	22.9 (3.5)	22.4 (3.9)	22.9 (3.4)	0.01
Medical history: stroke, %	4.0	7.3	3.5	<0.001
Medical history: diabetes, %	13.4	13.2	13.4	0.92
Medical history: hypertension, %	42.5	42.4	42.5	1.00
Frailty, %	31.2	58.9	27.7	<0.001
PA, %				
Low (<2.5 METs-h/week)	38.0	57.6	35.4	<0.001
Moderate (2.5–16.0 METs-h/week)	37.7	31.4	38.5	
High (>16.0 METs-h/week)	24.3	11.0	26.0	
MASB, reading time, %				
Low (<10 min/day)	22.3	27.0	21.6	0.02
Moderate (10–30 min/day)	48.7	46.0	49.0	
High (>30 min/day)	29.1	27.0	29.3	
PSB, TV-viewing time, %				
Low (<1 h/day)	19.0	17.9	19.1	0.05
Moderate (1–3 h/day)	52.6	49.3	53.1	
High (>3 h/day)	28.4	32.8	27.8	

MASB, mentally active sedentary behavior; PA, physical activity; PSB, passive sedentary behavior.

### Incidence of dementia for each category of physical activity and sedentary behaviors

**Figure 2.**
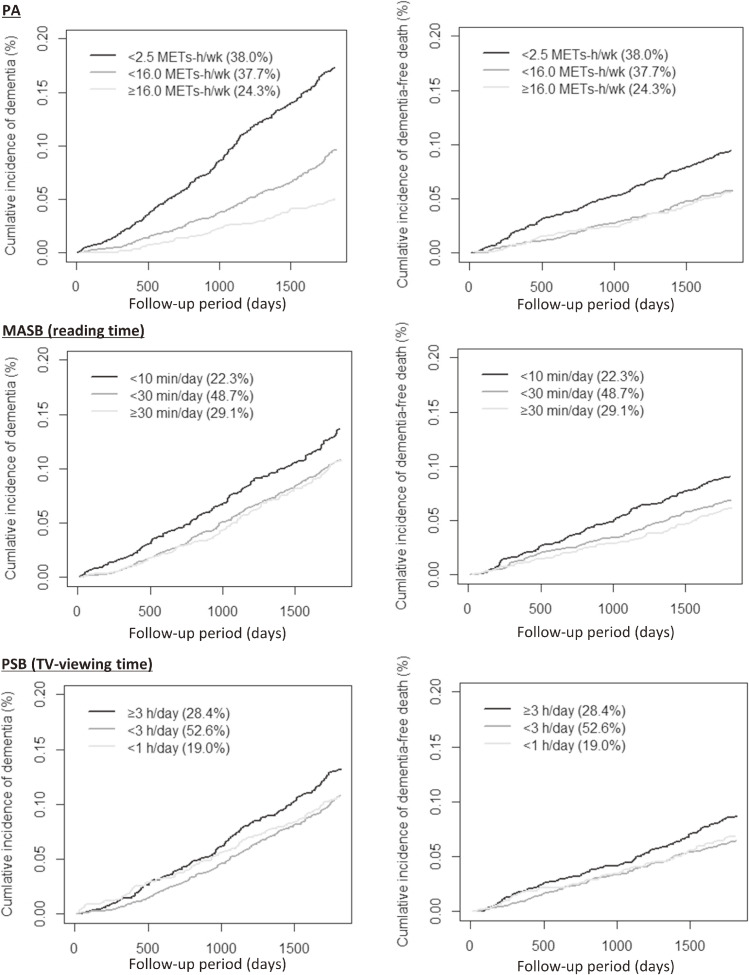
Cumulative incidence function of follow-up time to dementia with dementia-free death as a competing risk, by physical activity (PA) level, mentally active sedentary behavior (MASB), and passive sedentary behavior (PSB).

The cumulative incidence of dementia and dementia-free deaths during the 5-year study is shown in Figure 2 and in eTable 1. The cumulative incidence of dementia was the highest in the low-PA category (17.5%; 95% CI, 15.7–19.0), the low-MASB category (13.8%; 95% CI, 12.0–15.9), and the high-PSB category (13.7%; 95% CI, 12.0–15.5) (Figure 2 and eTable 2).

### The independent impacts of physical activity and sedentary behavior on dementia onset

The associations of each behavior with the incidence of dementia are shown in Table 2. A dose-response relationship for PA was observed, where both moderate- and high-PA categories were associated with a lower hazard of dementia onset. The high-MASB group was associated with a decreased dementia risk compared with the low-MASB group. There was no association between PSB and dementia risk (Table 2).

**Table 2.** The independent impact of physical activity and mentally active and passive sedentary behaviors on dementia onset (*n* = 5,323)

	%	Incidence per 1,000 person-years (95% CI)	Model 1	Model 2
	
			sdHR (95% CI)	sdHR (95% CI)
**PA**				
Low (<2.5 METs-h/week)	38.0	39.8 (35.8–44.2)	Reference	Reference
Moderate (2.5–16.0 METs-h/week)	37.7	20.3 (17.6–23.5)	0.78 (0.63–0.96)	0.72 (0.59–0.89)
High (>16.0 METs-h/week)	24.3	10.8 (8.4–13.8)	0.57 (0.42–0.77)	0.52 (0.38–0.70)

**MASB (reading time)**				
Low (<10 min/day)	22.3	31.2 (26.6–36.4)	Reference	Reference
Moderate (10–30 min/day)	48.7	23.5 (20.8–26.4)	0.83 (0.67–1.03)	0.83 (0.67–1.03)
High (>30 min/day)	29.1	22.8 (19.5–26.6)	0.77 (0.61–0.98)	0.75 (0.59–0.95)

**PSB (TV-viewing time)**				
Low (<1 h/day)	19.0	23.5 (19.3–28.4)	Reference	Reference
Moderate (1–3 h/day)	52.6	23.2 (20.6–26.0)	1.00 (0.82–1.22)	0.98 (0.80–1.19)
High (>3 h/day)	28.4	29.4 (25.5–33.8)	1.12 (0.86–1.45)	1.05 (0.81–1.37)

CI, confidence interval; MASB, mentally active sedentary behavior; PA, physical activity; PSB, passive sedentary behavior; sdHR, subdistribution hazard ratio.

Model 1: Fine-Gray model. Dependent variable: dementia onset; Independent variables: physical activity, reading time, TV-viewing time; Covariates: sex, age, years of education, marital status, living status, employment status, self-rated health, body mass index, medical treatment (stroke, diabetes, hypertension), and frailty.

Model 2: Additional adjustments for other independent variables.

### Differences in the association of mentally active and passive sedentary behavior with dementia onset by physical activity level

The associations of MASB and PSB with dementia onset for each PA category are shown in Table 3. For MASB, although point estimates of sdHR were less than 1, its associations with dementia risk were nonsignificant in the low- and moderate-PA groups. In contrast, moderate and high MASB levels were associated with a lower sdHR for dementia development in the high-PA group.

**Table 3.** The associations between mentally active and passive sedentary behaviors and dementia onset for each physical activity level (*n* = 5,323)

	MASB (reading time)		

	Low (<10 min/day)	Moderate (10–30 min/day)	High (>30 min/day)
	
		sdHR (95% CI)	sdHR (95% CI)
**PA**			
Low (<2.5 METs-h/week)	Reference	0.89 (0.68–1.17)	0.75 (0.54–1.03)
	(11.7%)^a^	(17.6%)^a^	(8.7%)^a^
Moderate (2.5–16.0 METs-h/week)	Reference	0.80 (0.52–1.21)	0.77 (0.49–1.22)
	(7.1%)^a^	(19.1%)^a^	(11.6%)^a^
High (>16.0 METs-h/week)	Reference	0.44 (0.22–0.85)	0.46 (0.23–0.93)
	(3.5%)^a^	(12.1%)^a^	(8.7%)^a^

	PSB (TV-viewing time)		

	Low (<1 h/day)	Moderate (1–3 h/day)	High (>3 h/day)
	
		sdHR (95% CI)	sdHR (95% CI)

**PA**			
Low (<2.5 METs-h/week)	Reference	1.06 (0.82–1.38)	1.02 (0.72–1.44)
	(11.7%)^a^	(18.8%)^a^	(7.4%)^a^
Moderate (2.5–16.0 METs-h/week)	Reference	0.77 (0.54–1.11)	1.21 (0.76–1.91)
	(10.7%)^a^	(20.3%)^a^	(6.8%)^a^
High (>16.0 METs-h/week)	Reference	1.23 (0.65–2.32)	0.74 (0.27–2.02)
	(6.0%)^a^	(13.5%)^a^	(4.8%)^a^

CI, confidence interval; MASB, mentally active sedentary behavior; PA, physical activity; PSB, passive sedentary behavior; sdHR, subdistribution hazard ratio.

Fine-Gray model. Dependent variable: dementia onset; Independent variables: physical activity, reading time, TV-viewing time; Covariates: sex, age, years of education, marital status, living status, employment status, self-rated health, body mass index, medical treatment (stroke, diabetes, hypertension), frailty, and other independent variable; Competing risk: dementia-free death.

^a^Prevalence of each category group.

PSB, however, had no significant impact on dementia onset at any PA level (Table 3).

### The joint impact of physical activity and sedentary behavior on dementia onset

**Figure 3.**
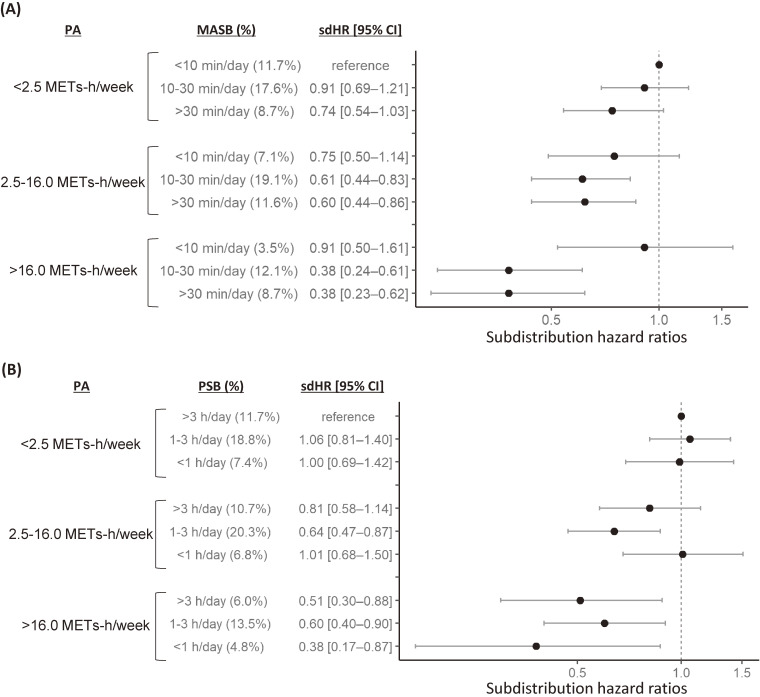
The joint impact of physical activity (PA) and mentally active (MASB) and passive sedentary behavior (PSB) on dementia onset. (**A**) Association of the combination of PA and MASB (reading time) with the risk of dementia; (**B**) Association of the combination of PA and PSB (TV-viewing time) with the risk of dementia.

The associations between the combinations of PA and MASB or PSB and dementia onset are shown in Figure 3. For the combination of PA and MASB, when PA was low, none of the MASB levels showed a significant difference in the risk of dementia compared with the reference (ie, low PA and low MASB). When PA was moderate or high, moderate and high MASB showed significantly lower sdHRs for dementia onset, but low MASB showed no significant difference. The combinations of high PA and moderate/high MASB showed the lowest sdHR: a 60% reduced risk of dementia.

For the combination of PA and PSB, none of the PSB levels showed a significant difference in the low-PA groups; moderate PSB showed a significantly lower sdHR in the moderate-PA groups. In the high-PA groups, dementia risk was significantly lower, irrespective of PSB level (Figure 3).

eTable 2, eTable 3, eTable 4, and eTable 5 indicate the results of the sensitivity analyses, including the models that excluded participants who developed dementia within 1 year from the baseline, and the models that used the data from baseline to the date of the state of emergency. Overall, the sdHRs of the analyses were generally similar to the main results (eTable 3, eTable 4, eTable 5, and eTable 6).

## DISCUSSION

This study aimed to examine the associations between dementia onset and MASB and PSB and determine whether these associations differ by PA level. The results indicated that only a high level of MASB was associated with lower dementia risk, but there was no association between PSB and dementia onset. The combinations of high PA and moderate/high MASB showed a reduced risk of dementia onset but no combined effect between PA and PSB. Our findings suggest that high MASB has a suppressive effect on dementia onset, and its impact is increased by combining it with high PA levels. These findings can help develop multicomponent interventions to prevent dementia onset.

### The independent impact of sedentary behavior on dementia onset

MASB and PSB showed different impacts on dementia onset; high MASB had a more suppressive impact than low MASB, but PSB showed no impact. The same results have been reported in a previous study. Lee and colleagues reported that intellectual activity, including reading books and newspapers, had a protective effect on the development of dementia, while recreational activity, including watching TV, did not affect dementia onset.^38^ Although the reason for the differing impacts of MASB and PSB on dementia onset is not entirely clear, depressive symptoms may mediate MASB’s association with dementia. Depression in later life is a significant risk factor for dementia,^45^ and MASB is associated with a lower risk of depression.^12^^,^^46^ Additionally, MASB can improve cognitive reserves because of the high cognitive demands it imposes.^47^ Those with greater cognitive reserves can tolerate more neuropathology and delay dementia onset.^48^

The nonsignificant association between PSB and dementia risk may be attributable to the relatively short follow-up period. Previous studies have identified a negative association between TV-viewing time and cognitive function when the follow-up period lasts more than 5 years.^19^^,^^20^ Therefore, based on that finding, 5 years of follow-up may be inadequate to detect a negative relationship between PSB and dementia onset.

### Differences in the association between sedentary behavior and dementia onset by physical activity level

Among the impacts of MASB on dementia in each PA category, we observed that moderate and high MASB in high-PA groups lowered dementia risk more than low MASB. These results indicate that the high level of PA had an additive cognitive effect to that of MASB. A previous meta-analysis suggested that combined physical and cognitive interventions do not have additive cognitive effects beyond cognitive training^49^; however, most of the studies included in the meta-analysis provided only low amounts of exercise. Potential mechanisms underlying the association have been suggested. PA and MASB may differentially affect cognitive function: while PA preserves neuronal structural integrity and brain volume, MASB improves neural circuit functioning and plasticity.^50^ These PA-induced structural changes, then, may enhance the effects of MASB on cognitive function. Another possible explanation for the association is that physically active individuals tend to have rich social networks, providing various opportunities to meet and communicate with people.^51^ These opportunities could contribute to acquiring new knowledge and skills through reading books or newspapers. Learning new things demands greater cognitive activity, which increases cognitive reserves even in later life.^47^^,^^48^ These potential mechanisms can help explain how high PA levels appear to induce additive effects in the independent impact of MASB on dementia onset. Further studies are needed to examine these potential mechanisms.

We did not observe a significant association between PSB and dementia onset at any PA level. This suggests that PA does not have an additive effect in the impact of PSB on dementia incidence.

### The joint impact of physical activity and sedentary behavior on dementia onset

Compared to the reference group, which combined low PA and low MASB, moderate and high MASB combined with moderate or high PA showed a significantly lower dementia risk. The combination of >16.0 METs-h/week of PA (eg, 30–40 min/day of MPA) and >10 min/day of MASB may reduce the risk of dementia by approximately 60%. Previous studies have suggested that multicomponent interventions would be necessary for an optimal preventive effect on dementia.^25^ The combination of PA and cognitive training is one efficacious approach.^49^ However, these programs were provided under supervision; they require staff members and facilities. These factors may be or create barriers to dissemination in the community setting. Lifestyle interventions that promote PA and daily MASB, by contrast, can be performed without supervision, which requires fewer professional workers and is less expensive. Therefore, delivering such programs to community-dwelling older adults is a feasible option for dementia prevention at the community level.

The dementia risk for those who performed high PA and low MASB was nonsignificant compared with the reference group. This may be because the prevalence of this group was low (3.5%). Additionally, the group might contain vision-impaired older adults or individuals living in poverty; we did not adjust for these variables. Further studies will be needed to detect the association.

When PA and PSB were combined, dementia risks in the high-PA groups were significantly lower than when PSB and low PA were combined, regardless of PSB level. Increasing PA and reducing SB is effective in preventing negative health outcomes, such as cardiovascular diseases or all-cause mortality.^52^ In dementia prevention, however, the preventive effects may depend on the amount of PA, not SB.

### Strengths and limitations

The strengths of this study are as follows. First, to reduce potential bias, the impacts of MASB and PSB on dementia onset were evaluated using a method that accounts for death as a competing risk. Second, the response rate to the survey was high (79.8%) enough to provide valid representation of the whole population of older adults in the survey area. Our findings, therefore, can be viewed as less biased than previous studies, and can usefully contribute to the development of interventions to prevent dementia.

This study has several limitations. First, PA and SB were self-reported, which may lead to overestimating.^53^ In the measurement of SB, we only assessed reading time (as MASB) and TV-viewing time (as PSB). Given that SB includes a wider variety of behaviors,^12^ future studies should examine the impacts of other indicators of MASB and PSB on dementia. Second, dementia onset evaluation was based on LTCI data. Participants who had developed dementia but had not applied for LTCI were not classified as “dementia onset.” Additionally, the actual dates of symptoms and the determined date of dementia onset might be different. Third, a longer follow-up period could be necessary to exclude the possibility of reverse causation. Fourth, there may have been residual confounding variables, such as genetic factors and other health behaviors. Fifth, because we conducted the survey in a rural area of Japan, variations in regional characteristics were not examined; thus, the transportability of our findings might be limited.

### Conclusion

We identified that high levels of MASB are independently associated with a lower risk of dementia onset, and the magnitude of MASB’s impact differs by PA level. In contrast, there was no association between PSB and developing dementia across all PA levels. Furthermore, the risk of dementia for individuals whose PA levels were high and whose MASB levels were moderate or high was approximately 60% lower than that for individuals with low PA and low MASB. Therefore, providing interventions to promote daily MASB, which contributes to reducing the risk of dementia, and PA, which increases the effects of MASB on dementia incidence, would be beneficial in delaying or preventing dementia onset.

## SUPPLEMENTARY MATERIAL

eTable 1. The independent impact of physical activity and mentally active and passive sedentary behaviors on dementia onset by sex

eTable 2. Cumulative incidence of dementia during five years (*n* = 5,323)

eTable 3. The independent impact of physical activity and mentally active and passive sedentary behaviors on dementia onset (sensitivity analysis)

eTable 4. The associations between mentally active and passive sedentary behavior and dementia onset stratified by physical activity level (sensitivity analysis)

eTable 5. The joint associations of physical activity (PA) and mentally active sedentary behavior (MASB) with dementia onset (sensitivity analysis)

eTable 6. The joint associations of physical activity (PA) and passive sedentary behavior (PSB) with dementia onset (sensitivity analysis)
