# Supplementary material for: Do the Impacts of Mentally Active and Passive Sedentary Behavior on Dementia Incidence Differ by Physical Activity Level? A 5-year Longitudinal Study
Source: J Epidemiol. 2023 Aug 5;33(8):410–8. doi: 10.2188/jea.JE20210419 (PMC10319527; doi:10.2188/jea.JE20210419)
Supplement: Supplementary file 1 [file je-33-410-s001.pdf]

eTable 1. The independent impact of physical activity and mentally active and passive sedentary behaviors on dementia onset by sex

		Fine-Gray model
	**%**	**sdHR (95% CI)**
**Males (n=2,421)**		
**PA**		
Low (<2.5 METs-h/week)	34.7	Reference
Moderate (2.5─16.0 METs-h/week)	33.9	0.81 (0.59–1.10))
High (>16.0 METs-h/week)	33.1	0.51 (0.34–0.78)
**MASB (reading time)**		
Low (<10 min/day)	21.0	Reference
Moderate (10─30 min/day)	47.0	0.83 (0.59–1.17)
High (>30 min/day)	32.0	0.71 (0.49–1.04)
**PSB (TV-viewing time)**		
Low (<1 h/day)	20.5	Reference
Moderate (1─3 h/day)	51.2	0.93 (0.69–1.24)
High (>3 h/day)	28.3	0.88 (0.60–1.30)
**Females (n=2,902)**		
**PA**		
Low (<2.5 METs-h/week)	40.6	Reference
Moderate (2.5─16.0 METs-h/week)	41.0	0.69 (0.54–0.90)
High (>16.0 METs-h/week)	18.3	0.55 (0.35–0.88)
**MASB (reading time)**		
Low (<10 min/day)	23.3	Reference
Moderate (10─30 min/day)	50.1	0.82 (0.61–1.10)
High (>30 min/day)	26.6	0.77 (0.55–1.07)
**PSB (TV-viewing time)**		
Low (<1 h/day)	17.7	Reference
Moderate (1─3 h/day)	53.8	1.02 (0.78–1.33)
High (>3 h/day)	28.5	1.16 (0.81–1.67)

CI, confidence interval; MASB, mentally active sedentary behavior; PA, physical activity; PSB, passive sedentary behavior; sdHR, subdistribution hazard ratio.
Fine-Gray model. Dependent variable: dementia onset; Independent variables: physical activity, reading time, TV-viewing time; Covariates: sex, age, years of education, marital status, living status, employment status, self-rated health, body mass index, medical treatment (stroke, diabetes, hypertension), and frailty.

eTable 2. Cumulative incidence of dementia during five years (*n*=5,323)

		Cumulative incidence of dementia onset		Cumulative incidence of dementia-free death	
		2.5 years	5 years	2.5 years	5 years
	%	% (95% CI)	% (95% CI)	% (95% CI)	% (95% CI)
**Physical activity**					
Low (<2.5 METs-h/week)	38.0	7.5 (6.3–8.6)	17.3 (15.7–19.0)	4.9 (3.9–5.8)	9.3 (8.0–10.5)
Moderate (2.5–16.0 METs-h/week)	37.7	3.1 (2.3–3.8)	9.5 (8.3–10.9)	2.8 (2.1–3.5)	6.0 (4.9–7.0)
High (>16.0 METs-h/week)	24.3	1.9 (1.1–2.6)	5.6 (4.4–7.0)	2.2 (1.4–3.0)	5.6 (4.3–6.8)
**Mentally active sedentary behavior (reading time)**					
Low (<10 min/day)	22.3	6.1 (4.8–7.5)	13.8 (12.0–15.9)	5.0 (3.7–6.2)	9.3 (7.6–10.9)
Moderate (10–30 min/day)	48.7	4.3 (3.5–5.1)	10.9 (9.8–12,2)	3.3 (2.6–3.9)	6.8 (5.9–7.8)
High (>30 min/day)	29.1	3.3 (2.4–4.2)	10.6 (9.1–12.2)	2.6 (1.8–3.4)	5.9 (4.8–7.1)
**Passive sedentary behavior (TV-viewing time)**					
High (>3 h/day)	28.4	5.4 (4.3–6.6)	10.5 (8.8–12.6)	3.3 (2.2–4.4)	6.9 (5.3–8.4)
Moderate (1–3 h/day)	52.6	4.0 (3.2–4.7)	10.6 (9.5–11.8)	3.1 (2.4–3.7)	6.3 (5.4–7.2)
Low (<1 h/day)	19.0	4.3 (3.0–5.5)	13.7 (12.0–15.5)	4.2 (3.2–5.3)	8.9 (7.5–10.3)

CI, confidence interval; MET, metabolic equivalent of task.

eTable 3. The independent impact of physical activity and mentally active and passive sedentary behaviors on dementia onset (sensitivity analysis)

	%	Incidence per 1000 person-years (95% CI)	Model 1 sdHR 95% CI)	Model 2 sdHR 95% CI)
**Analysis excluding participants who developed dementia within one year (n=5,257)**				
**Physical activity**				
Low (<2.5 METs-h/week)	37.5	34.5 (30.8–38.7)	Reference	Reference
Moderate (2.5-16.0 METs-h/week)	37.9	18.6 (15.9–21.5)	0.80 (0.64–0.99)	0.74 (0.60–0.92)
High (>16.0 METs-h/week)	24.5	10.5 (8.1–13.4)	0.62 (0.48–0.83)	0.55 (0.41–0.76)
**Mentally active sedentary behavior (reading time)**				
Low (<10 min/day)	22.1	26.6 (22.4–31.4)	Reference	Reference
Moderate (10-30 min/day)	48.8	21.2 (18.7–24.0)	0.86 (0.68–1.08)	0.86 (0.68–1.09)
High (>30 min/day)	29.1	20.9 (17.7–24.5)	0.80 (0.62–1.03)	0.78 (0.61–1.00)
**Passive sedentary behavior (TV-viewing time)**				
Low (<1 h/day)	18.9	19.8 (16.0–24.3)	Reference	Reference
Moderate (1-3 h/day)	52.8	21.1 (18.7–23.8)	1.01 (0.82–1.24)	0.99 (0.81–1.22)
High (>3 h/day)	28.4	26,2 (22.5–30.4)	1.04 (0.78–1.38)	0.99 (0.75–1.31)
**Analysis using the data before the state of emergency (n=5,323)**				
**Physical activity**				
Low (<2.5 METs-h/week)	38.0	37.6 (33.4–42.2)	Reference	Reference
Moderate (2.5-16.0 METs-h/week)	37.7	17.3 (14.6–20.5)	0.74 (0.59–0.94)	0.67 (0.60–0.92)
High (>16.0 METs-h/week)	24.3	10.6 (8.0–13.8)	0.65 (0.47–0.90)	0.57 (0.41–0.76)
**Mentally active sedentary behavior (reading time)**				
Low (<10 min/day)	22.3	28.4 (23.7–33.7)	Reference	Reference
Moderate (10-30 min/day)	48.7	22.2 (19.4–25.3)	0.87 (0.69–1.11)	0.86 (0.68–1.10)
High (>30 min/day)	29.1	20.4 (17.1–24.3)	0.78 (0.59–1.03)	0.73 (0.61–0.96)
**Passive sedentary behavior (TV viewing time)**				
High (>3 h/day)	28.4	28.1 (23.9–32.7)	Reference	Reference
Moderate (1-3 h/day)	52.6	20.9 (18.3–23.7)	0.94 (0.76–1.18)	0.92 (0.74–1.15)
Low (<1 h/day)	19.0	21.5 (17.2–26.6)	1.08 (0.81–1.44)	1.01 (0.76–1.35)

CI, confidence interval; sdHR, subdistribution hazard ratio.

Model 1: Fine-Gray model including dementia onset as dependent variable, one of physical activity, reading time, TV-viewing time as independent variable, sex, age, years of education, marital status, living status, employment status, self-rated health, body mass index, medical treatment (stroke, diabetes, hypertension), and frailty as covariates, dementia-free death as competing risk.

Model 2: Model 1 and other independent variables as covariate.

eTable 4. The associations between mentally active and passive sedentary behavior and dementia onset stratified by physical activity level (sensitivity analysis)

	Mentally active sedentary behavior (Reading time)		
	**Low (<10 min/day)**	**Moderate (10–30 min/day)**	**High (>30 min/day)**
		**sdHR (95% CI)**	**sdHR (95% CI)**
**Analysis excluding participants who developed dementia within one year (n=5,257)**			
**Physical activity**			
Low (<2.5 METs-h/week)	Reference (11.4%) [a]	0.98 (0.73–1.33) (17.4%) [a]	0.81 (0.57–1.14) (8.7%) [a]
Moderate (2.5–16.0 METs-h/week)	Reference (7.1%) [a]	0.75 (0.48–1.17) (19.1%) [a]	0.75 (0.47–1.22) (11.7%) [a]
High (>16.0 METs-h/week)	Reference (3.5%) [a]	0.44 (0.22–0.87) (12.2%) [a]	0.45 (0.22–0.91) (8.8%) [a]
**Analysis using the data before the state of emergency (n=5,323)**			
**Physical activity**			
Low (<2.5 METs-h/week)	Reference (11.7%) [a]	0.96 (0.71–1.29) (17.6%) [a]	0.78 (0.54–1.09) (8.7%) [a]
Moderate (2.5–16.0 METs-h/week)	Reference (7.1%) [a]	0.77 (0.48–1.26) (19.1%) [a]	0.70 (0.42–1.19) (11.6%) [a]
High (>16.0 METs-h/week)	Reference (3.5%) [a]	0.50 (0.23–1.05) (12.1%) [a]	0.51 (0.23–1.12) (8.7%) [a]

	Passive sedentary behavior (TV-viewing time)		
	**High (>3 h/day)**	**Moderate (1–3 h/day)**	**Low (<1 h/day)**
		**sdHR (95% CI)**	**sdHR (95% CI)**
**Analysis excluding participants who developed dementia within one year (n=5,257)**			
**Physical activity**			
Low (<2.5 METs-h/week)	Reference (11.6%) [a]	1.10 (0.83–1.46) (18.7%) [a]	0.93 (0.63–1.36) (7.2%) [a]
Moderate (2.5–16.0 METs-h/week)	Reference (10.7%) [a]	0.79 (0.54–1.14) (20.4%) [a]	1.17 (0.72–1.90) (6.8%) [a]
High (>16.0 METs-h/week)	Reference (6.1%) [a]	1.19 (0.62–2.26) (13.6%) [a]	0.76 (0.28–2.06) (4.9%) [a]
**Analysis using the data before the state of emergency (n=5,323)**			
**Physical activity**			
Low (<2.5 METs-h/week)	Reference (11.7%) [a]	0.94 (0.70–1.26) (18.8%) [a]	0.99 (0.68–1.44) (7.4%) [a]
Moderate (2.5–16.0 METs-h/week)	Reference (10.7%) [a]	0.73 (0.48–1.12) (20.3%) [a]	1.18 (0.71–1.98) (6.8%) [a]
High (>16.0 METs-h/week)	Reference (6.0%) [a]	1.38 (0.66–2.90) (13.5%) [a]	0.56 (0.13–2.36) (4.8%) [a]

CI, confidence interval; sdHR, subdistribution hazard ratio.

Fine-Gray model included dementia onset as dependent variable, reading time/TV-viewing time as independent variable, sex, age, years of education, marital status, living status, employment status, self-rated health, body mass index, medical treatment (stroke, diabetes, hypertension), frailty, and TV-viewing time/reading time as covariates, dementia death as semi-competing risk.

[a] Prevalence of each category group.

eTable 5. The joint associations of physical activity (PA) and mentally active sedentary behavior (MASB) with dementia onset (sensitivity analysis)

	Analysis excluding participants who developed dementia within one year (n=5,257)		Analysis using the data before the state of emergency (n=5,323)	
**Combination of PA and MASB**	**%**	**sdHR (95% CI)**	**%**	**sdHR (95% CI)**
Low PA and Low MASB	11.4	Reference	11.7	Reference
Low PA and Moderate MASB	17.4	1.00 (0.74–1.36)	17.6	0.97 (0.72–1.31)
Low PA and High MASB	8.7	0.82 (0.58–1.15)	8.7	0.76 (0.54–1.08)
Moderate PA and Low MASB	7.1	0.85 (0.55–1.32)	7.1	0.74 (0.46–1.20)
Moderate PA and Moderate MASB	19.1	0.65 (0.46–0.92)	19.1	0.59 (0.42–0.84)
Moderate PA and High MASB	11.7	0.67 (0.47–0.96)	11.6	0.60 (0.37–0.82)
High PA and Low MASB	3.5	1.03 (0.58–1.84)	3.5	0.94 (0.50–1.76)
High PA and Moderate MASB	12.2	0.43 (0.27–0.70)	12.1	0.44 (0.27–0.73)
High PA and High MASB	8.8	0.43 (0.26–0.72)	8.7	0.42 (0.24–0.74)

CI, confidence interval; sdHR, subdistribution hazard ratio.

Fine-Gray model included dementia onset as dependent variable, reading time/TV-viewing time as independent variable, sex, age, years of education, marital status, living status, employment status, self-rated health, body mass index, medical treatment (stroke, diabetes, hypertension), frailty, and TV-viewing time/reading time as covariates, dementia death as semi-competing risk.

PA: low indicates <2.5 metabolic equivalent of task [MET]s-h/week, moderate indicates 2.5–16.0 METs-h/week, high indicates >16.0 METs-h/week.

MASB: low indicates <10 min/day, moderate indicates 10–30 min/day, high indicates >30 min/day.

eTable 6. The joint associations of physical activity (PA) and passive sedentary behavior (PSB) with dementia onset (sensitivity analysis)

	Analysis excluding participants who developed dementia within one year (n=5,257)		Analysis using the data before the state of emergency (n=5,323)	
**Combination of PA and PSB**	**%**	**sdHR (95% CI)**	**%**	**sdHR (95% CI)**
Low PA and High PSB	11.6	Reference	11.7	Reference
Low PA and Moderate PSB	18.7	1.11 (0.83–1.47)	18.8	0.94 (0.71–1.26)
Low PA and Low PSB	7.2	0.90 (0.63–1.36)	7.4	0.96 (0.66–1.40)
Moderate PA and High PSB	10.7	0.85 (0.60–1.21)	10.7	0.72 (0.50–1.05)
Moderate PA and Moderate PSB	20.4	0.66 (0.47–0.92)	20.3	0.52 (0.37–0.75)
Moderate PA and Low PSB	6.8	1.01 (0.66–1.54)	6.8	0.90 (0.58–1.40)
High PA and High PSB	6.1	0.56 (0.32–0.99)	6.0	0.49 (0.27–0.89)
High PA and Moderate PSB	13.6	0.63 (0.42–0.97)	13.5	0.65 (0.43–0.99)
High PA and Low PSB	4.9	0.39 (0.17–0.89)	4.8	0.26 (0.08–0.86)

CI, confidence interval; PA, physical activity; PSB, passive sedentary behavior; sdHR, Subdistribution hazard ratio.

Fine-Gray model included dementia onset as dependent variable, reading time/TV-viewing time as independent variable, sex, age, years of education, marital status, living status, employment status, self-rated health, body mass index, medical treatment (stroke, diabetes, hypertension), frailty, and TV-viewing time/reading time as covariates, dementia death as semi-competing risk.

PA: low indicates <2.5 metabolic equivalent of task [MET]s-h/week, moderate indicates 2.5–16.0 METs-h/week, high indicates >16.0 METs-h/week.

PSB: low indicates <1 h/day, moderate indicates 1–3 h/day, high indicates >3 h/day.
